# Adolescents With Eating Disorders in Pediatric Practice – The European Academy of Paediatrics Recommendations

**DOI:** 10.3389/fped.2022.806399

**Published:** 2022-04-26

**Authors:** Agnieszka Rynkiewicz, Łukasz Dembiński, Berthold Koletzko, Pierre-André Michaud, Adamos Hadjipanayis, Zachi Grossman, Kathryn Korslund, Bryan H. King, Janet Treasure, Jarosław Peregud-Pogorzelski, Stefano del Torso, Arunas Valiulis, Artur Mazur

**Affiliations:** ^1^Department of Psychiatry, College of Medical Sciences, Institute of Medical Sciences, University of Rzeszow, Rzeszow, Poland; ^2^Center for Diagnosis, Therapy and Education SPECTRUM ASC-MED, Gdańsk, Poland; ^3^The European Academy of Paediatrics (EAP), Brussels, Belgium; ^4^Department of Pediatric Gastroenterology and Nutrition, Medical University of Warsaw, Warsaw, Poland; ^5^Department of Paediatrics, Dr. von Hauner Children’s Hospital, University of Munich Medical Centre, Ludwig-Maximilians-Universität Munich, Munich, Germany; ^6^Faculté de Biologie et de Médecine, Université de Lausanne, Lausanne, Switzerland; ^7^School of Medicine, European University Cyprus, Nicosia, Cyprus; ^8^Department of Paediatrics, Larnaca General Hospital, Larnaca, Cyprus; ^9^Adelson School of Medicine, Ariel University, Ariel, Israel; ^10^Maccabi Health Services, Tel Aviv, Israel; ^11^THIRA Health, Bellevue, WA, United States; ^12^Department of Psychiatry and Behavioral Sciences, Weill Institute for Neurosciences, University of California, San Francisco, San Francisco, CA, United States; ^13^Eating Disorder Unit, Institute of Psychiatry, Psychology and Neuroscience, King’s College London, London, United Kingdom; ^14^Department of Pediatrics, Pediatric Oncology and Immunology, Pomeranian Medical University, Szczecin, Poland; ^15^Polish Society of Paediatrics, Warsaw, Poland; ^16^ChildCare WorldWide-CCWWItalia OdV, Padova, Italy; ^17^Clinic of Children’s Diseases, Institute of Clinical Medicine, Vilnius University Medical Faculty, Vilnius, Lithuania; ^18^Department of Public Health, Institute of Health Sciences, Vilnius University Medical Faculty, Vilnius, Lithuania; ^19^Department of Paediatrics, College of Medical Sciences, Institute of Medical Sciences, University of Rzeszow, Rzeszow, Poland

**Keywords:** anorexia nervosa, binge-eating disorder, bulimia nervosa, early diagnosis, primary care

## Abstract

In the face of the growing number of adolescents suffering from eating disorders (EDs) and access to psychiatric care limited by the epidemiological and demographic situation, the primary care pediatrician’s role in diagnosing and treating EDs is growing. The European Academy of Paediatrics (EAP) decided to summarize knowledge about EDs and formulate recommendations to support European pediatricians and improve care for adolescents with EDs.

## Introduction

Pediatricians commonly encounter patients with suspected or confirmed eating disorders (EDs), who carry a considerable risk of morbidity and even mortality. Early detection, appropriate diagnostic workup, and effective treatment require transdisciplinary collaboration. They are all essential for improving the patients’ prognosis. Here we aim to review current knowledge relevant for practicing pediatricians and provide recommendations of the European Academy of Paediatrics (EAP) on the role of pediatricians in caring for adolescents with ED and for actions of authorities and pediatric societies aiming to improve healthcare for these patients.

## Classification

The Diagnostic and Statistical Manual of Mental Disorders, fifth edition (DSM-5) and the 11th revision of the International Classification of Diseases (ICD-11) define ED as persistent abnormal eating behaviors that significantly impair physical health or psychosocial functioning (1, 2). The diagnostic criteria and characteristics of the most common ED in the adolescent population – anorexia nervosa (AN), bulimia nervosa (BN), binge-eating disorder (BED), avoidant/restrictive food intake disorder (ARFID) – are presented in Table 1 (2).

**TABLE 1:** Diagnostic criteria for selected eating disorders (1, 3).

Eating disorder	Diagnostic criteria
Anorexia nervosa (AN)	• Restriction of energy intake leading to a bodyweight significantly below minimally expected for age and sex.
	• Intense fear of gaining weight.
	• Disturbance of body mass and shape perception, excessive influence of body weight or shape on self-value.
Bulimia nervosa (BN)	• Recurrent episodes of binge eating characterized by: eating, in a discrete period of time, definitely larger amount of food that is than what most individuals would eat in a similar period of time under similar circumstances and a sense of lack of control over eating during the episode.
	• Recurrent inappropriate compensatory behaviors to prevent weight gain (e.g., fasting, excessive exercise, self-induced vomiting, misuse of laxatives, or diuretics).
	• The binge eating and compensatory behaviors both occur at least once a week for 3 months.
	• Excessive influence of body weight or shape on self-value.
	• The binge eating and compensatory behaviors do not occur exclusively during episodes of AN.
Binge-eating disorder (BED)	• Recurrent episodes of binge eating characterized by: eating, in a discrete period of time, definitely larger amount of food that is than what most individuals would eat in a similar period of time under similar circumstances and a sense of lack of control over eating during the episode.
	• The binge-eating episodes include three or more of the following: eating much more quickly than usual, eating until uncomfortably full, eating large amounts of food when not feeling hungry, eating alone because of embarrassment at how much one is eating, and feeling guilty, disgusted, or depressed afterward.
	• Marked distress regarding binge eating is present.
	• The binge eating occurs at least once a week for 3 months.
	• The binge eating is not associated with the recurrent use of inappropriate compensatory behavior as in BN and does not occur exclusively during BN or AN.
Avoidant/restrictive food intake disorder (ARFID)	• An eating or feeding disturbance (e.g., apparent lack of interest in eating or food; avoidance based on the sensory characteristics of food; concern about aversive consequences of eating) as manifested by persistent failure to meet appropriate nutritional and/or energy needs associated with at least one of the following: significant weight loss or failure to achieve expected growth and/or weight gain, marked nutritional deficiency, reliance on enteral feeding, or oral nutritional supplements, significant interference with psychosocial functioning.
	• The eating disturbance does not occur exclusively during AN or BN, and there is no evidence of disturbance of body mass and shape perception.
	• The disturbance cannot be better explained by lack of available food, an associated culturally sanctioned practice, or another mental disorder/coexisting medical condition.

Other less common EDs include:

• Atypical AN – AN with normal body weight;
• Rumination-regurgitation disorder – repeated regurgitation of food not associated with other medical conditions;
• Pica – persistent eating of non-nutritive/non-food substances;
• Purging disorder – recurrent purging behavior to influence weight or shape (e.g., vomiting, misuse of laxatives or diuretics, or other medications) in the absence of BED.

In practice, one type of ED may shift to another over time, and in some patients, it is difficult to diagnose a particular type of ED precisely.

## Epidemiology

As the onset of the disease is usually in the teenage years, pediatricians are the most likely to suspect a diagnosis of ED (4, 5). Data on the prevalence of ED are quite varied and depend on the population and diagnostic criteria used. Large-scale population surveys also have an underestimation bias due to patients’ tendency to minimize symptoms or deny the disease (6). However, the overall prevalence of ED is estimated from 2% to even 6%, with a nearly five times greater frequency of BED than other types of ED (7–10). The COVID-19 pandemic also contributed to a significant increase in stress, disturbances in school and family relations, and often limited access to psychological counseling, which increased the frequency of ED and deterioration of the prognosis (11).

Statistically, ED are more common in females, although in BED, the proportion of male patients is higher approximately a third (12).

Episodes of behavior typical of ED but not meeting all criteria may occur in up to one in five adolescents (13). However, some behaviors may mask or mimic ED at this age, which is mainly related to the adolescent gaining influence on their own nutrition and focusing attention on external appearance (14). Autism spectrum disorder shares some common features with ED, especially the restrictive ones, which should be considered in the differential diagnosis (15, 16). ED can also coexist with other mental or organic disorders, such as depression, celiac disease, or diabetes (17).

A specific cause of ED has not been established, but potential etiology includes genetic predisposition, cognitive and emotional vulnerability, social and environmental factors, such as pressure of the dominant cultural patterns and weight stigma (18). Moreover, in the group of children with ED, there are increased risks of emotional or behavioral disorders, attention-deficit/hyperactivity disorder and autism spectrum disorders (19, 20).

It should also be noted that an increased risk of ED occurs in children struggling with the lack of social acceptance (e.g., overweight, ethnic, racial, or sexual minorities) (21, 22).

## Impact on Health

Eating disorder should be seen not only as a mental illness but as a threat to health and even to life. Chronic malnutrition, nutritional deficiencies, frequent vomiting, and misuse of drugs can lead to irreversible effects that impair adolescents’ health and development. Chronic undernutrition can lead to long-term effects, such as diminished bone density or even cognitive impairment (23). Moreover, the consequences of ED can cause secondary peer acceptance problems, bullying, symptoms of post-traumatic stress disorder, and an increased risk of self-injury (24–26). ED has the second-highest mortality rate of all mental health disorders due to the increased frequency of suicide attempts but also due to the health consequences of cachexia (e.g., electrolyte problems, dehydration, heart failure) (27, 28).

The main health problems that adolescents with ED may report to a pediatrician are summarized in Table 2.

**TABLE 2:** Major health complaints, symptoms, and signs in adolescents with eating disorders.

General	Significant loss or inadequacy of body weight, growth failure, fatigue, obesity, hypothermia, episodes of fainting, or loss of consciousness
Psychiatric	Depressed mood, sleep disturbances, anxiety
Gastrointestinal	Dysphagia, constipation, laxative dependence, gastroesophageal reflux, esophagitis (Mallory–Weiss syndrome)
Cardiological	Bradycardia, low blood pressure, cardiac murmurs
Dermatological	Hair loss, pallor, dry skin, lanugo, Russell’s sign, bruising over the spine
Endocrinologic	Amenorrhea, delayed puberty
Hematologic	Anemia, leukopenia, thrombocytopenia
Dental	Dental enamel erosions, fetor ex ore
Renal	Polyuria, nocturia
Skeletal	Osteopenia, osteoporosis

## Screening and Early Diagnosis

Due to the fatal consequences of untreated ED, early diagnosis and therapeutic intervention improve outcomes. Awareness of the various ED symptoms and screening in risk groups is an essential role of the primary care pediatrician and nurse. Vaccination visits and annual health check-ups provide a unique opportunity to spot the first signs of ED. Therefore, assessing the nutritional status with reference to growth charts at each visit is essential (29).

Apart from the findings in the physical examination described in Table 2, blood pressure and heart rate should be assessed. Lowering these values in relation to age references may be a symptom of cachexia and (e.g., in the case of significant bradycardia) indicate the need for hospitalization.

In the ED risk assessment, several available tools can be used. To date, many different physician-filled or self-assessed screening questionnaires, which can help to detect abnormal behaviors and lead to earlier diagnosis, have been developed, e.g.:

• SCOFF questionnaire – containing five short questions about eating and its impact on life (30);
• Eating disorder Screen for Primary care (ESP) – containing questions about eating patterns and previous ED episodes (31);
• Eating Disorders Assessment for DSM-5 (EDA-5) – containing questions based on the DSM-5 diagnosis criteria (32);
• Child Eating Disorder Examination (ChEDE) – containing 28 questions to assess diagnostic criteria for BED and BN (33);
• Bright Futures Questionnaires – a relatively extensive questionnaire from the American Academy of Pediatrics containing questions about eating patterns and body image (34).

However, the results of questionnaires, especially those self-reported, should be interpreted with caution and in the context of the entire clinical picture. Due to the lack of an unequivocal advantage of one questionnaire, pediatricians should use the one with the interpretation of which they have the most experience and is available in the national language.

The primary care pediatrician can also directly ask questions about, e.g., inducing vomiting, using laxatives, or self-image, but it is essential to maintain an atmosphere of respect and trust. A comprehensive psychosocial assessment is also necessary, emphasizing the possibility of coexistence of addictions, bullying, and physical or sexual abuse (35, 36).

Laboratory tests should be considered in patients with suspected ED to exclude anemia, disturbances of electrolyte equilibrium, thyroid dysfunction, and celiac disease – Table 3. However, the diagnosis of ED is purely clinical, and diagnostic procedures mentioned above mainly aim to identify metabolic and physiological disturbances that are consequences of ED. Among patients with severe cachexia, cardiological evaluation (electrocardiogram and echocardiogram) is vital to exclude cardiac arrhythmias and pericardial effusion (37). Some patients may require additional radiological, gastroenterological, endocrinological, or neurological workup to rule out causes of symptoms other than ED.

**TABLE 3:** Recommended diagnostics in patients with suspected ED in primary pediatric care.

General	Body weight, height, BMI – with reference to growth charts, blood pressure, heart rate
Hematological	Total blood count, ferritin, CRP
Endocrinological	TSH
Gastroenterological	IgA, tissue transglutaminase antibodies (tTG-IgA)
Cardiological	Electrocardiogram (ECG)

## Treatment

Most adolescents with ED can be treated in an outpatient setting provided that a patient and family are motivated to cooperate, and there are no severe malnutrition, somatic complications, or other psychiatric problems requiring hospitalization (38). In such cases, cooperation between a psychiatrist and a psychologist with a primary care pediatrician is essential to optimize treatment strategies and ensure patient safety. In addition, since treatment of EDs requires an interdisciplinary approach, dietitians, family therapists, social workers, and teachers, whenever possible, should be involved in treating patients with ED.

Family based treatment is the recommended front-line treatment for ED in childhood and adolescents. However, Cognitive Behavior Therapy or its modification – Dialectical Behavior Therapy – can be a useful supplement (39). However, in addition, regular monitoring of the patient’s nutritional status by the primary care pediatrician is vital especially in cases of AN.

Hospitalization should be considered in adolescents who develop life-threatening complications of ED or have failed to restore weight with outpatient treatment. Effective hospital treatment needs a professional, multidisciplinary team with expertise and experience dealing with ED patients and a structured treatment plan that is regularly re-evaluated and revised when necessary. In addition, patients with coexisting mental and organic diseases, pericardial effusion, electrolyte disorders, and a high risk of developing the refeeding syndrome require special supervision (40, 41).

Pharmacotherapy (e.g., antidepressants or neuroleptics) is also used in the treatment of ED, but it should be carried out with the leading role of a psychiatrist.

Pediatricians should consider that, as with many chronic diseases, ED affects the patient’s entire family. For example, many parents can be depressed, grieve, or express guilt because of their child’s illness (42). Also, siblings may develop their own psychological and psychiatric problems (43).

Early diagnosis and adequate treatment can produce a partial remission in over 60% of patients (44, 45).

## The Role of Pediatricians

Primary care pediatricians play a crucial role in screening and early diagnosis of ED because they have the unique opportunity to monitor adolescents’ health regularly, and they have the chance, to some extent, assess adolescents’ family and social situations. Therefore, a mandatory part of the examination should be the assessment of nutritional status, as it may be the first sign of ED.

The role of pediatricians is also to educate and inform the community about ED and advocate for these patients. Moreover, in the face of the shortage of child psychiatrists in many European countries, the pediatrician often will have to act as the treatment coordinator (46).

## Recommendations

For international and national state, health, and education authorities

• Adolescents with ED require multidisciplinary care. Therefore, this issue should be present in undergraduate and postgraduate medical education.
• National health authorities should provide adolescents with ED with multidisciplinary care and support for their families.

For European and national pediatric societies

• Pediatric societies should support their members through ongoing training in ED.
• Pediatric societies should advocate in increasing the availability of psychiatric and psychological care for adolescents with ED.

For pediatricians

• An integral part of each patient visit should be the assessment of their nutritional status.
• Measurement of blood pressure and heart rate should be a part of every physical examination of adolescents with ED.
• When assessing the risk of ED, pediatricians should use the questionnaires with which they have the most experience and are available in the local language.
• The role of a pediatrician is to cooperate with a psychiatrist and psychologist in the therapeutic process, with particular emphasis on regular assessment of the nutritional status and early identification of health risk signs.
• Adolescents with life-threatening complications of ED and those who have failed in weight normalization efforts should be referred to the hospital.

## Data Availability Statement

The original contributions presented in the study are included in the article, further inquiries can be directed to the corresponding author.

## Author Contributions

AR, ŁD, BK, AH, and AM: study design. AR and ŁD: data collection. AR, ŁD, BK, and P-AM: data analysis and interpretation. AR, ŁD, BK, P-AM, AH, ZG, KK, BHK, JT, JP-P, ST, AV, and AM: manuscript preparation and critical revision. All authors read and approved the final manuscript.

## Conflict of Interest

The authors declare that the research was conducted in the absence of any commercial or financial relationships that could be construed as a potential conflict of interest.

## Publisher’s Note

All claims expressed in this article are solely those of the authors and do not necessarily represent those of their affiliated organizations, or those of the publisher, the editors and the reviewers. Any product that may be evaluated in this article, or claim that may be made by its manufacturer, is not guaranteed or endorsed by the publisher.
